# Redox Homeostasis and Nrf2-Regulated Mechanisms Are Relevant to Male Infertility

**DOI:** 10.3390/antiox13020193

**Published:** 2024-02-03

**Authors:** Cinzia Signorini, Luciano Saso, Somayyeh Ghareghomi, Pelin Telkoparan-Akillilar, Giulia Collodel, Elena Moretti

**Affiliations:** 1 Department of Molecular and Developmental Medicine, University of Siena, 53100 Siena, Italy; cinzia.signorini@unisi.it (C.S.); giulia.collodel@unisi.it (G.C.); elena.moretti@unisi.it (E.M.); 2 Department of Physiology and Pharmacology “Vittorio Erspamer”, Sapienza University of Rome, 00185 Rome, Italy; 3 Institute of Biochemistry and Biophysics, University of Tehran, Tehran 1417466191, Iran; ghareghomi.s@ut.ac.ir; 4 Department of Medical Biology, Faculty of Medicine, Yuksek Ihtisas University, 6530 Ankara, Turkey; pelinta@yiu.edu.tr

**Keywords:** human male infertility, nuclear factor-E2-related factor 2 (Nrf2), oxidative stress, sperm

## Abstract

Infertility represents a significant global health challenge, affecting more than 12% of couples worldwide, and most cases of infertility are caused by male factors. Several pathological pathways are implicated in male infertility. The main mechanisms involved are driven by the loss of reduction–oxidation (redox) homeostasis and the resulting oxidative damage as well as the chronic inflammatory process. Increased or severe oxidative stress leads to sperm plasma membrane and DNA oxidative damage, dysregulated RNA processing, and telomere destruction. The signaling pathways of these molecular events are also regulated by Nuclear factor-E2-related factor 2 (Nrf2). The causes of male infertility, the role of oxidative stress in male infertility and the Keap1-Nrf2 antioxidant pathway are reviewed. This review highlights the regulatory role of Nrf2 in the balance between oxidants and antioxidants as relevant mechanisms to male fertility. Nrf2 is involved in the regulation of spermatogenesis and sperm quality. Establishing a link between Nrf2 signaling pathways and the regulation of male fertility provides the basis for molecular modulation of inflammatory processes, reactive oxygen species generation, and the antioxidant molecular network, including the Nrf2-regulated antioxidant response, to improve male reproductive outcomes.

## 1. Introduction

Infertility is described as the inability to conceive after one year or more of orderly unprotected sexual intercourse. Available data estimates that 8–12% of couples worldwide face fertility problems [1]. Traditionally, it was thought that reproductive issues concern only women, mainly due to cultural beliefs, while nowadays, it is well recognized that fertility and, as a result, infertility are dependent on the couple. Infertility may be due to male factors, female factors, or a combination of them, or it can be defined as sine causa or idiopathic [2].

In half of the cases, there is a male infertility aspect independently or in grouping with a female aspect [3].

Oxidative stress (OS) is introduced as a common cause of male infertility by disrupting the structural and functional integrity of spermatozoa. Physiological level of reactive oxygen species (ROS) is essential for sperm capacitation and acrosomal reaction; in contrast, the pathological levels of ROS damage sperm quality [4]. DNA is one of the most oxidatively affected molecules; in particular, ROS overproduction can lead to sperm DNA fragmentation, caused by a mismatch of bases, loss of base, base modifications, DNA adducts and crosslink, pyrimidine dimers compromising natural and assisted fertilization outcomes [5,6].

Nuclear factor-E2-related factor 2 (Nrf2) is an endogenous antioxidant factor which controls a collection of genes to prevent redox imbalances in cells. Based on various studies, Nrf2 expression in sperm from patients affected by oligospermia was significantly lower than that in healthy men, signifying that Nrf2 was critical in spermatogenesis. Therefore, the modulatory effect of Nrf2 as a master antioxidant pathway is important in male fertility rate [7,8].

This review provides a summary of the pathophysiology of OS-induced harm in human sperm and its consequences for male fertility. The modulatory effect of the Nrf2 antioxidant signaling pathway on male fertility is then discussed.

### A Brief General Overview of Male Infertility

Generally, causes of male infertility can be identified as congenital and acquired factors acting at pre-testicular, post-testicular or directly at the testicular level. Among congenital factors, anorchia, cryptorchidism and genetic abnormalities such as karyotype anomalies and chromosome microdeletions can be mentioned [9]. Acquired factors include testis trauma, testicular torsion, obstruction of the proximal or distal urogenital tract, varicocele, recurrent urogenital infections, and inflammations, hypogonadotropic hypogonadism or endocrine factors, exogenous and environmental factors such as smoking, alcohol, drug intake, occupational exposure to toxins, pollution [1].

It is noteworthy that the reason of about 30% [10] of cases of male infertility remains unclear and unexplained and it is ascribed to idiopathic infertility. Research in this case is mandatory.

Male gametes are produced in the seminiferous tubules by a process known as spermatogenesis [11] by which spermatogonia develop into mature spermatozoa with the contribution of different cell types, hormones, paracrine factors, and gene expression and epigenetics pathways. In addition to the germ line, spermatogenetic process is orchestrated by somatic cells. The peritubular myoid cells surround the seminiferous tubules and show contractile properties necessary to help the propulsion of testicular fluid for the transport of spermatozoa in the tubule. Leydig cells are present in the interstitial tissue and underpin the life-long androgen production. Both kinds of cells are target for hormones, cytokines, and growth factors, and they produce many substances necessary to support spermatogenesis. However, the real key driver of testis function is the Sertoli cells [12] that physically support and interact with all the germ lines in the seminiferous epithelium. They play a role in regulating androgen production, in arranging the blood–testis barriers, in cross-talking with the other somatic cells involved in spermatogenesis. This complex cell framework necessary to support a normal spermatogenesis can be target of OS, which impairs testicular and epididymal functions, and can affect the prostate and seminal vesicles, the major producers of seminal plasma.

Phenotypic manifestation of male infertility may range from the total absence of spermatozoa in the seminiferous tubules to different alterations of sperm quality. The World Health Organization (WHO) Laboratory Manual for the Examination and Processing of Human Semen [2] provides standard laboratory methods for semen analysis that are extensively used both in clinical practice and for research and allows internationally comparable results [13].

The semenogram is a fast, simple, and economical test that requires well-trained examiners and little equipment; however, its diagnostic and prognostic value for reproductive purposes is moderate, and evaluation of semen parameters such as sperm concentration, motility and morphology is not sufficient to predict reproductive success [14].

For this reason, there is a need to develop additional and advanced functional tests that complement and enrich the semenogram data providing information about sperm physiology [15] and mechanisms by which the different reproductive pathologies can affect male infertility.

**Figure 1:**
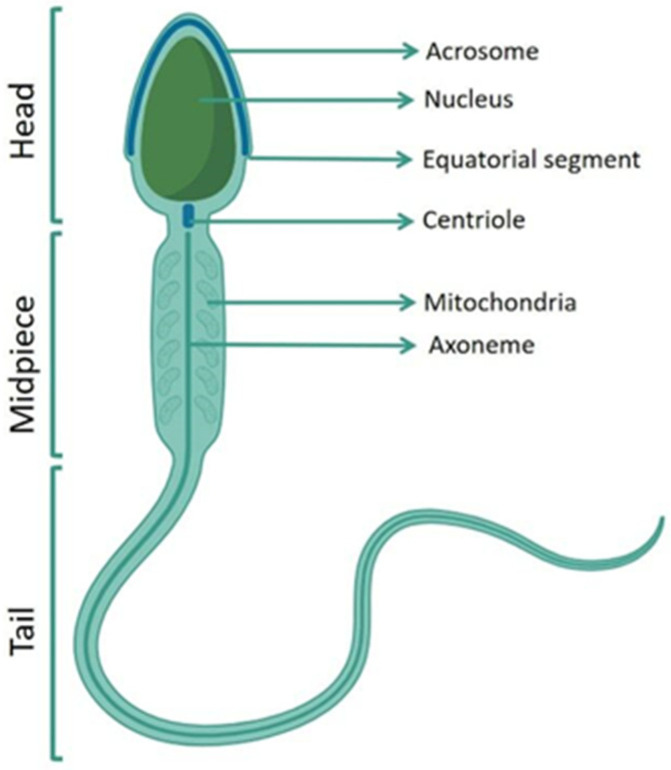
A schematic human spermatozoon. Human spermatozoon comprises three main sections: head, midpiece, and tail. The structures that characterize each region are indicated. The acrosome is located in the apical part of the nucleus and contains lytic enzymes; the nucleus is characterized by highly condensed chromatin; the equatorial segment is located at the end of acrosome. From the centriole the axoneme originates, the mitochondria surround the axoneme in the midpiece.

These tests allow the examination of the individual “functional compartments” of the spermatozoon such as membrane, acrosome, chromatin, mitochondria and flagellum (Figure 1), the interaction of spermatozoon–oocyte and biomarkers found in the semen.

Llavanera et al. [15] recently reviewed the literature on some Omics, such as genomics and epigenomics, transcriptomics, and proteomics, and concluded that DNA structure and integrity can be considered as relevant molecular biomarkers that may help in the diagnosis and treatment of conditions causing semen quality disorders and fertilization failure. In addition, metabolomics and in particular ROS evaluation in spermatozoa and seminal plasma, total antioxidant capacity and oxidation–reduction potential have been proposed as well-characterized and promising molecular fertility biomarkers in semen with potential clinical application [15].

## 2. Male Infertility and Oxidative Stress

A disturbance in the signaling of redox network results in an imbalance in the redox steady state, leading to a disruption of homeostasis and the occurrence of OS. This condition favors the oxidative damages with respect to the antioxidant defenses; subsequently, it causes damage to biomolecules through oxidative reactions [16]. As oxidants, oxygen free radicals and additional ROS are known to be involved both in physiological and pathological (oxidative damage) mechanisms [17,18,19]. In sperm, ROS play a relevant role in regulating several intracellular pathways. Because ROS generation influences sperm membrane fluidity, activation of adenylate cyclase, and protein tyrosine phosphorylation, the normal physiological ROS levels mediate sperm capacitation, the acquisition of hyperactivated motility, the acrosome reaction, and the sperm–oocyte interaction [20,21].

High levels of ROS can cause infertility not only by lipid peroxidation or DNA oxidative damage, but also by inactivation of enzymes and oxidation of proteins in spermatozoa [22], consequently affecting sperm quality and impairing male fertility [23,24]. In human ejaculate, ROS originate mainly from immature spermatozoa and leukocytes [25,26,27].

Spermatozoa are particularly vulnerable to oxidative harm because of the abundant presence of polyunsaturated fatty acids (PUFA) in plasma membranes and the limited capacity to mend damage caused by ROS. Seminal plasma contains a significant amount of antioxidants such as glutathione peroxidase (GPx), catalase (CAT), and superoxide dismutase (SOD) [28] that help maintain the redox equilibrium under normal physiological circumstances; also, sperm cells contain antioxidants, but at extremely low concentrations [19]. Nevertheless, in some conditions, the production of ROS increases, overpowering the antioxidant activity, leading to OS [29].

ROS measurement in seminal fluid, which is an important element in the evaluation of male fertility, has also become an important target in preconception care [30].

Lipid peroxidation, which involves the oxidative damage of lipids, proceeds through three distinct phases (initiation, propagation and termination) along which free radicals are first produced, then quantitatively increased and finally neutralized by antioxidants or through reactions between free radicals [31]. In sperm, the lipid vulnerability to oxidative damage leads to the accumulation of lipid peroxides within spermatozoa, resulting in the formation of various decay end-products, including 4-hydroxynonenal, isoprostanes, and malondialdehyde (MDA). Such lipid peroxidation end-products serve as measurable indicators of OS [32]. There are many studies showing that lipid peroxidation of the sperm plasma membrane, as the main consequence of OS, is associated with poor sperm quality and infertility [33,34]. In sperm membranes, fatty acids with unstable bonds are vulnerable to oxidation by ROS.

In reproductive tissues, linoleic acid (LA), alpha-linolenic acid (ALA), their metabolites eicosapentaenoic acid (EPA), docosahexaenoic acid (DHA), and arachidonic acid (ARA), may influence reproductive function and fertility. In the sperm plasma membrane, lipid composition and specific types of PUFA (n-3 PUFA or n-6 PUFA) are related to membrane fluidity, flexibility, and receptor function. Ultimately, sperm membrane composition in PUFA highly regulates sperm membrane fusion at the time of sperm–oocyte interaction. In sperm, lipid composition and membrane lipid metabolism contribute to the formation of membrane microdomains involved in sperm motility, capacitation, and ability of sperm to penetrate the zona pellucida [35,36].

Dietary fats can influence the lipid composition of sperm cells, having harmful or beneficial consequences on male reproductive potential. An investigation is currently underway to explore the biochemical mechanisms that modulate sperm quality through diet [37,38,39,40].

Male infertility is a complex issue that can arise from various factors, including the interconnected pathophysiological processes of OS and inflammation.

These common mechanisms play a significant role in the development of male infertility, highlighting the need for further research in this area [41].

OS has been advocated in male infertility [42] associated with main pathologies altering the male reproductive system [43,44], bacterial infections, inflammation and leukocytospermia, environmental and lifestyle factors, and chronic/degenerative diseases [42,45,46,47]. In particular, in chronic prostatitis, OS plays an important role not only affecting male fertility, but also influencing the prostate cancer development. Large amounts of ROS and pro-inflammatory cytokines are continuously produced when inflammation persists. Consequently, this pathway triggers the activation of transcription factor nuclear factor-kappa B (NF-κB) and genes encoding for further production of pro-inflammatory cytokines, chemotactic factors, and growth factors [48].

Inflammation has been associated with many reproductive pathological conditions [49,50,51,52]. Increased levels of inflammatory cytokines, leukocyte counts that affect sperm quality and inflammation are also associated with sperm necrosis. It was recently observed that low sperm quality and sperm necrosis positively correlated with iron metabolism indices (ferritin, iron, transferrin evaluated in seminal plasma) in individuals with leukocytospermia and varicocele, both of which are characterized by the presence of inflammatory status and OS condition [53]. Altered iron metabolism is involved in ROS production [54,55,56,57] and drives chronic inflammation affecting male fertility [58,59]. Interestingly, several studies reported that ferroptosis, an iron-dependent programmed cell death, can affect spermatogenetic process leading to male infertility [60,61].

On this topic, it has been reported that pro-resolving lipid mediators, which are able to regulate the resolution of acute inflammation, are related to male infertility [62]. Interestingly, such pro-resolving lipid mediators are derived from PUFA. Mainly, pro-resolving lipid mediators of inflammation are biosynthesized from PUFA precursors, e.g., ARA, EPA, DHA, and docosapentaenoic acid [63].

## 3. Keap1-Nrf2 Antioxidant Pathway

**Figure 2:**
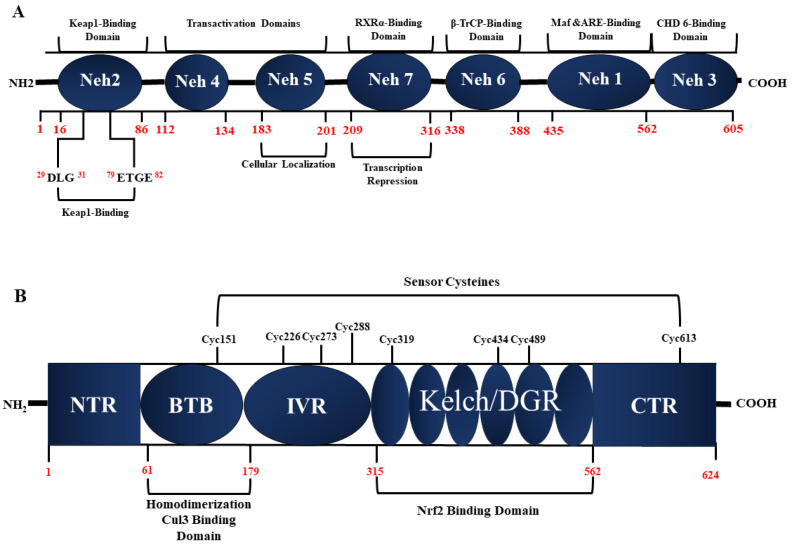
The structural domains of Nrf2 (**A**) and Keap1 (**B**). (**A**): Nrf2 comprises seven Nrf2-ECH homeodomains (Neh). For each Neh domain, the specific activity is indicated. (**B**): Keap1 has five domains, including NTR (N-terminal region), BTB (Bric a brac, tramtrack and broad complex), IVR (intervening region), Kelch (Kelch or DGR domains) and CTR (C-terminal region). The specific role is indicated for each region. The amino acid residues for each domain are indicated in red.

The Keap1-Nrf2 antioxidant pathway is a crucial cellular defense mechanism that plays a pivotal role in protecting organisms from oxidative stress and maintaining redox homeostasis. This intricate signaling pathway regulates the activation of transcription factor Nrf2 which, in turn, orchestrates the expression of several antioxidant and detoxification genes. The central element of this pathway is Kelch-like ECH associating protein 1 (Keap1). Under basal conditions, Keap1 directs Nrf2 for ubiquitin-dependent degradation, thereby repressing Nrf2-dependent gene expression. Under oxidative stress conditions, the Nrf2 level increases, leading to the activation of Nrf2-dependent gene expression [64]. The Nrf2 protein (Figure 2A), spanning 605 amino acids, is organized into seven highly conserved regions identified as Nrf2-ECH homology (Neh) domains [65]. These Neh domains (Neh1-7) play critical roles in regulating6 Nrf2’s stability and transcriptional activity (transactivation). Specifically, the Neh2 domain engages with two Keap1 molecules through the ETGE and DLG motifs, with the interaction and stability of Nrf2 being governed by Neh2.

Neh5 regulates the cellular localization of Nrf2, ensuring its proper positioning within the cell [66,67] The Neh1 domain, beyond controlling Nrf2 stability through its basic leucine zipper motif, facilitates the binding of Nrf2 to the antioxidant response element (ARE) sequence. This unique role in antioxidant responses induced by the pathway accentuates Neh1’s significance [68].

The Neh6 domain regulates Keap1-independent degradation of Nrf2, introducing an additional layer of complexity. The activation of ARE-dependent genes, post-chromatin remodeling, occurs following the interaction of Neh3 with the transcriptional co-activator CHD6, a DNA-binding protein with chromo-ATPase/helicase activity. Transcription activation domains Neh4 and Neh5 bind to the co-activator cAMP-responsive element-binding protein, facilitating Nrf2 transcription [69]. Lastly, the Neh7 domain, through interaction with retinoic X receptor α, can repress Nrf2 [70]. These sophisticated interactions and regulatory mechanisms within the various Neh domains underscore the complexity of the Keap1-Nrf2 antioxidant pathway, elucidating its pivotal role in cellular responses to oxidative stress.

Human Keap1, composed of 624 amino acid residues, boasts a molecular weight of approximately 70 kD [71]. As depicted in Figure 2B, this protein showcases five distinct domains: three broad complex–tramtrack–bric-a-brac (BTB) domains, one intervening region (IVR), and two glycine repeat domains (DGR). Each of these domains plays a pivotal role in inhibiting the activity of Nrf2 [72]. In a Keap1 homodimer, the DGR domains bind to different affinities with the DLG and ETGE domains in a single Nrf2 molecule. Under oxidative conditions, the separation of the DLG motif of Nrf2 from the DGR domain in Keap1 hinders Nrf2 degradation [73].

The IVR domain in Keap1 contains a consensus sequence of a nuclear export signal, crucial for Keap1’s cytoplasmic localization and its interaction with the Cul3 protein. Cul3, in conjunction with Roc1, constitutes the E3 ligase complex responsible for ubiquitinating Nrf2 [74,75]. Keap1 acts as a sensor for various stress stimuli, with numerous cysteine residues serving as critical sensing points. Notably, Cys273 and Cys288 are essential for Keap1 to regulate Nrf2 under both basal and stress conditions, while Cys151 is primarily required under stress conditions [76,77]. Other cysteine residues in Keap1, including Cys226, Cys434, and Cys613, play crucial roles in sensing specific toxins [73,78]. Modification of these cysteine residues inhibits the ubiquitin E3 ligase activity of the Keap1-Cul3 complex [79]. The intricacies of Keap1’s structural organization and the precise tuning of its cysteine residues underscore its role as a sophisticated cellular sensor and regulator in the Keap1-Nrf2 antioxidant pathway.

The induction of Nrf2 in response to OS leads to its release from Keap1, triggering its translocation to the nucleus. Upon reaching the nucleus, Nrf2 undergoes dimerization with small musculoaponeurotic fibrosarcoma oncogene homolog (Maf) proteins. This resulting Nrf2-Maf complex exhibits selective targeting towards genes possessing ARE in their regulatory regions. These genes constitute a diverse array of crucial components, including antioxidant enzymes, ABC transporters, and various stress-responsive proteins [79].

The Nrf2-Maf complex, selectively directed towards ARE-containing genes, plays a pivotal role in orchestrating a robust cellular defense against OS. Activation of the Nrf2 pathway induces the expression of a spectrum of antioxidant enzymes, including SOD, CAT, GPx, quinone oxidoreductase-1 (NQO1), and heme oxygenase-1 (HO-1). These enzymes collectively contribute to the modulation of oxidative conditions, neutralizing ROS and counteracting the detrimental effects of OS [80,81].

**Figure 3:**
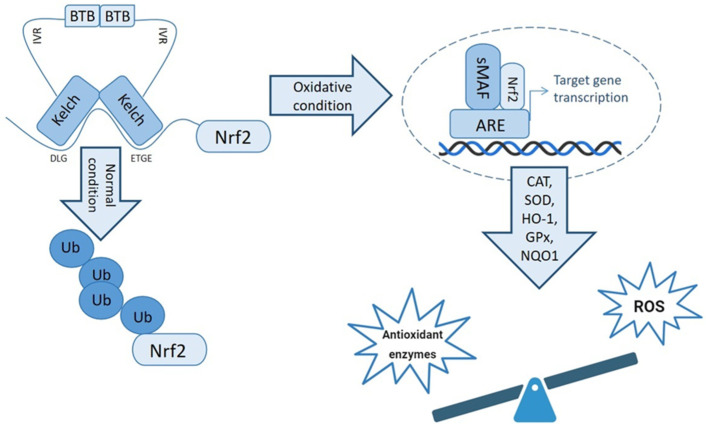
The Nrf2 antioxidative pathway. Activation of the Nrf2 transcription factor in oxidative conditions led to upregulation of Nrf2-related gene expression and reduction in reactive oxygen species (ROS). In normal conditions, ubiquitination of Nrf2 leads to proteasomal degradation. ARE, antioxidant response element; Nrf2, nuclear erythroid-factor-2; SOD, Superoxide Dismutase; CAT, Catalase; GPx, Glutathione Peroxidase, NQO1, quinone oxidoreductase-1; HO-1, heme oxygenase-1; sMAF, small musculoaponeurotic fibrosarcoma oncogene homolog, Ub: ubiquitin.

The Nrf2 antioxidant pathway, as summarized in Figure 3, serves as a critical mechanism in the cellular response to OS. By activating a repertoire of antioxidant enzymes and stress-responsive proteins, Nrf2 contributes significantly to the maintenance of cellular redox homeostasis and the prevention of oxidative damage. Understanding the intricacies of Nrf2-mediated responses provides valuable insights into potential therapeutic strategies for conditions associated with oxidative stress.

## 4. Role of Nrf2 in Male Fertility

Nrf2 appears to be a key regulator of the molecular mechanisms involved in male infertility. According to various studies, the Nrf2 function seems to play a beneficial role in safeguarding spermatogenesis against an excessive amount of ROS, thereby preserving specific sperm functions, including motility, which may otherwise be compromised under conditions of elevated ROS exposure [82].

### 4.1. Oxidative Stress and Nrf2-Related Gene Regulation

Several studies have shown a link between sperm abnormalities (function and/or morphology) and OS. Since Nrf2 is a transcription factor regulating the cellular response to stress conditions, its activity is involved in checking/regulating redox stress and inflammation. Thus, Nrf2 is a molecule of impressive relevance in male reproduction [83].

Since low Nrf2 levels as well as low antioxidant levels are associated with poor sperm quality, analyzing the transcripts (mRNA) of antioxidant genes is a fascinating issue. Interestingly, *NRF2* gene expression was found to be associated with SOD activity and gene expression for SOD and CAT in human spermatozoa with low motility [84]. A gene expression profile related to OS and coordinated by the Nrf2/Keap1 system has been reported in rat and human testis and epididymis [85]. Functional nucleotide polymorphisms have been identified in the *NRF2* promoter regions in idiopathic asthenozoospermic and oligoasthenozoospermic patients compared to the control group. These polymorphisms have also been related to altered levels of Nrf2 mRNA and/or decreased levels of antioxidant gene expression in spermatozoa [86]. In rat testis, OS results in the downregulation of the *Nrf2* gene and to amplified testicular lipid peroxidation, germ cell death, and diminution of antioxidant levels [87].

Additionally, it was shown that reduced NRF2 expression in mouse testis and human spermatozoa is linked to dysfunction of spermatogenesis in case of diabetes mellitus and a smoking habit [88,89].

Diabetes is described as a condition of hyperglycemia that induces OS deleterious for male fertility. Indeed, in patients with diabetes, extremely high levels of hydroxyl radicals and advanced glycation end-products, induced by hyperglycemia, can trigger the triad: generation of ROS, OS and low sperm quality.

Even in this case, Nrf2 plays a pivotal role in diabetic testes because the autophagy mechanism induced by ROS activates a pathway that suppress Nrf2 activation worsening oxidative damage [90]. It is interesting to consider that an eventual upregulation of Nrf2-Keap1 pathway can increase the ratio nuclear Nrf2/cytosolic Nrf2 committing the transcription of SOD, CAT and GPx mRNAs.

Also, in obesity, a condition strongly linked to male infertility via OS, apoptosis, and glycolysis [91], the PKA/ERK/Nrf2 signaling axis mediates the improvement in fertility [92]. Moreover, the Keap1/Nrf2/ARE signaling pathway, which plays a key role in the OS response, was activated in reproductive system diseases [93], and enhanced Nrf2 expression was involved in the reduction in OS in patients with obesity [94].

### 4.2. Effects of Nrf2 Gene Deletion on Spermatogenesis

In 2010, Nakamura et al. [82] generated *Nrf2* knockout (*Nrf2−/−*) mice by disrupting the gene through homologous recombination in embryonic stem cells. They observed that the absence of the transcription factor NRF2 caused a severe age-related spermatogenetic impairment. The effect of gene deletion was evident in mice by the age of 6 months in which sperm concentration decreased both in testis and epididymis, and the motility of epididymal spermatozoa was reduced with respect to those of wild-type mice. Concomitantly, the testes of *Nrf2−/−* male mice underwent an age-related process of declining in weights, indicating a process of degeneration. Furthermore, *Nrf2−/−* male mice were found to show elevated levels of testicular and epididymal lipid peroxidation, increased testicular germ cell apoptosis, and reduced antioxidant levels compared to wild-type animals [82]. This mouse model highlighted the crucial role played by transcription factor NRF2 in preventing oxidative disruption of spermatogenesis.

### 4.3. Thermoregulation Mechanisms of Nrf2 in Spermatogenesis

Nrf2 also appears to be involved in the thermoregulation mechanisms that regulate spermatogenesis. It is well known that spermatogenesis is influenced by temperature, and the elevation of testicular temperature can seriously affect mammalian spermatogenesis and the quality of semen [95] involving also somatic cells that are highly susceptible to elevated temperature [96]. Because of heat stress induced in mouse testes, the mRNA expression of Nrf2 increases together with the expression of different genes involved in redox homeostasis, promoting the expression of antioxidant genes [97].

He et al. [98] reported that in mice, Sertoli cells under heat stress activate the Keap1-Nrf2 pathway to prevent heat-induced oxidative damage.

### 4.4. Role of Nrf2 in Long-Term Storage of Sperm

By considering the supporting role of Nrf2 to spermatogenesis and sperm quality, Sadeghiani et al. [99] reported that pentoxifylline supplementation during freezing protocol and long-term storage protected mouse spermatogonial stem cell activating the antioxidant defense through the Nrf2/ARE signaling pathway. In addition, the transcription of *NRF2*, together with that referred to *SOD1* and *GPx1*, was up-regulated in post-thawed buck semen after supplementation with antioxidants [100].

Jannatifar et al. [101] supplemented the cryopreservation medium with *N*-acetyl-cysteine (NAC) and alpha lipoic acid, used during a freezing protocol of semen samples of patients with asthenoteratozoospermia. They found that both antioxidants protected spermatozoa increasing the expression level of *NRF2* gene and consequently the level of antioxidant enzymes, mainly CAT and SOD.

**Figure 4:**
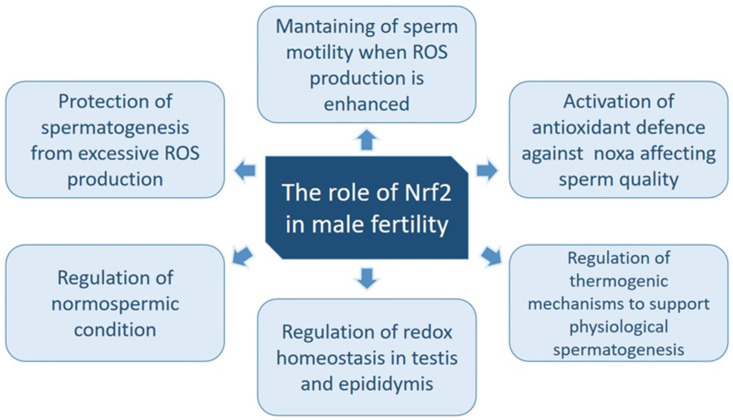
Nrf2 roles in male fertility. Nrf2 is a regulator of multiple mechanisms involved in the adequate production and maturation of sperm. The figure summarizes the main roles of Nrf2 in the regulation of male fertility. Further details and bibliographical references are given in Section 4.

In Figure 4, the roles of Nrf2 in spermatogenesis and sperm quality are summarized.

## 5. Compounds Targeting Nrf2 and Influencing Male Fertility

Several studies conducted in animal models or in humans focused on the use of different compounds that influence the Nrf2 pathway negatively or positively. Based on the mentioned roles of Nrf2 in the regulation of OS, its targeting can display a remarkable effect on various aspects of cell development and fertility.

It is well known that therapeutic drugs, especially chemotherapeutics, can affect male fertility [102,103,104]. Cyclophosphamide (CYP) is one of the most successful anti-cancer agents ever synthesized. It is commonly utilized as a chemotherapeutic compound and in the conditioning regimens for blood and marrow transplantation [105]. Despite the anticancer properties of this agent, its metabolite led to increase in OS and apoptosis in the testes and male infertility. Maremanda et al. [106] reported that in rats, the treatment with CYP induced a decrease in Nrf2 levels in the testicular tissue. Furthermore, CYP therapy disrupted Nrf2 downstream pathway molecules such as HO-1 and SOD and the physiological antioxidant response. The concomitant administration of zinc improved the damages caused by CYP by modulating several mechanisms included a Nrf2-associated pathway [106].

Hydroxytyrosol (HT), one of the most common phytochemicals existing in oil and table olives, exerts strong antioxidant capacity and potential to demolish free radicals [107,108,109]. Recently, Fusco et al. [110] tested, in mice, a new compound Hidrox^®^ based on HT as treatment for spermatogenetic damages induced by CYP. These authors found that the antioxidant capacities were not limited to the expression of type 2 detoxifying proteins such as SOD, CAT and GPx. Indeed, there are adaptive systems such as those involving HO-1, the expression of which is regulated by Nrf2.

Another tested compound, decursin, extracted from *Angelica gigas* Nakai roots, exerted an antioxidant activity involving Nrf2 in a cryptorchidism-induced infertility rat model. Cryptorchid rats treated with an *A. gigas* extract showed improved testis weight and sperm parameters in parallel with decreased OS and apoptosis and increased levels of Nrf2-related genes including SOD and HO-1 [111].

For this purpose, Arkali et al. [112] studied the protective effect of carvacrol, a phenolic monoterpene with antioxidant activity present in essential oils of aromatic plants, on diabetes-induced reproductive damage in male rats by evaluating the Nrf2/HO-1 pathway, NF-κB-mediated apoptosis and sperm parameters. They found that carvacrol significantly reduced MDA levels, Bax and NF-κB protein expression levels, and significantly increased Bcl-2, Nrf2/HO-1 levels and consequently GPx and CAT activities; this condition exerted a positive effect on sperm parameters. Other antioxidant compounds can target the Nrf2 pathway involved in redox homeostasis of male reproductive systems.

For example, treatment of rats exposed to cadmium [113] with piceatannol (PT; trans-3,4,3′,5′-tetrahydroxystilbene) present in various fruit and vegetables [114] protected the testis from alterations induced by the heavy metal. Spermatogenesis and steroidogenesis were improved, and concomitantly OS was reduced by targeting the Nrf2-Keap1 signaling pathway and its related genes.

Lycopene (LYC) is another effective antioxidant compound mostly present in tomatoes and other fruits with recognized health benefits [115]. Zhao et al. [116] demonstrated the beneficial effects of LYC in enhancing the expressions of Nrf2 and its downstream related genes and attenuating damages to Leydig cells of mice experimentally treated with di(2-ethylhexyl) phthalate, a plasticizer known to induce OS.

Melatonin, a hormone produced by the pineal gland, is involved in regulating biological timing. It is secreted at night in all species and is thereby associated with physiological events. This hormone, through its antioxidant properties, can modulate OS in various cells. In a mouse model of restraint stress, the administration of melatonin decreased the ROS level, amplified SOD and GSH activities, and downregulated iNOS and tumor necrosis factor-α (TNF-α) activities in testes. In addition, melatonin upregulated the expression of antioxidant proteins including Nrf2 and HO-1 with an improvement of spermatogenesis and sperm production [117].

In addition, melatonin inhibited the apoptosis of rooster Leydig cells in vitro by suppressing oxidative stress via AKT-Nrf2 pathway activation [118] and modulated Nrf2 activity to protect porcine pre-pubertal Sertoli cells from the abnormal H_2_O_2_ generation due to cadmium exposition [119].

Another tested antioxidant molecule is aucubin, extracted by *Eucommia ulmoides* and used as a protective treatment in mouse testis damaged by triptolide [120]. Aucubin, increasing the translocation of Nrf2 into the nucleus, triggered antioxidant enzymes in the testis, decreasing apoptotic process and protecting the blood–testis barrier integrity.

To the best of our knowledge, studies reporting data on the modulation of NRF2 male human reproduction are scant. Zhou et al. [121] treated in vitro spermatozoa from patients with asthenozoospermia with curcumin, a polyphenol with antioxidant activity, and observed that the mechanism of action by which curcumin exerted protective activity included the NRF2 pathway.

Additionally, as far as we know, only a couple of clinical trials in which the male subjects were treated with antioxidant compounds considered the effect on the NRF2 pathway. The treatment of infertile asthenozoospermic patients with NAC oral supplementation (600 mg, three times daily) improved sperm quality and decreased OS levels by enhancing *NRF2* gene expression [122]. In a different clinical trial, Fallahi et al. [123] treated 60 infertile patients with date palm pollen (DPP, 400 mg/kg), a natural dietary food supplement rich in bioactive unsaturated fatty acid and flavonoid compounds [124]. DPP has a positive effect on reducing ROS levels, increasing sperm parameters and expression of NRF2, GPx4, SOD2, and CAT in infertile men.

## 6. Conclusions and Future Perspective

High levels of OS result in damage to sperm parameters and ultrastructure, and therefore might provide a common underlying etiology of male infertility.

OS is predominantly caused by lifestyle-related factors, the majority of which are modifiable.

Upregulation of some antioxidative pathways can reverse this effect and exert a remarkable effect on increasing male fertility. Therefore, in addition to the detailed investigation of the causes of infertility, the use of effective antioxidants to stimulate the master antioxidative pathway, including Nrf2 and modulation of the ROS levels, can be considered as a healthy treatment method to increase fertility rate in males.

Furthermore, the stimulation of antioxidant pathways, the support of Nrf2-regulated mechanisms in cryopreservation procedures, might represent concrete supplementary in vitro fertilization techniques in humans and animals.

## Data Availability

No new data were created in this study. Data availability sharing is not applicable.
